# Does multiple paternity influence offspring disease resistance?

**DOI:** 10.1111/jeb.12854

**Published:** 2016-03-29

**Authors:** K. E. Thonhauser, S. Raveh, M. Thoß, D. J. Penn

**Affiliations:** ^1^ Konrad Lorenz Institute of EthologyDepartment of Integrative Biology and EvolutionUniversity of Veterinary MedicineViennaAustria; ^2^ Department of Environmental SciencesZoology and EvolutionUniversity of BaselBaselSwitzerland

**Keywords:** bet‐hedging, genetic diversity, multiple paternity, *Mus musculus*, pathogen resistance, pathogen‐mediated sexual selection, polyandry, salmonella, sex differences in immunity

## Abstract

It has been suggested that polyandry allows females to increase offspring genetic diversity and reduce the prevalence and susceptibility of their offspring to infectious diseases. We tested this hypothesis in wild‐derived house mice (*Mus musculus*) by experimentally infecting the offspring from 15 single‐ and 15 multiple‐sired litters with two different strains of a mouse pathogen (*Salmonella Typhimurium)* and compared their ability to control infection. We found a high variation in individual infection resistance (measured with pathogen loads) and significant differences among families, suggesting genetic effects on *Salmonella* resistance, but we found no difference in prevalence or infection resistance between single‐ vs. multiple‐sired litters. We found a significant sex difference in infection resistance, but surprisingly, males were more resistant to infection than females. Also, infection resistance was correlated with weight loss during infection, although only for females, indicating that susceptibility to infection had more harmful health consequences for females than for males. To our knowledge, our findings provide the first evidence for sex‐dependent resistance to *Salmonella* infection in house mice. Our results do not support the hypothesis that multiple‐sired litters are more likely to survive infection than single‐sired litters; however, as we explain, additional studies are required before ruling out this hypothesis.

## Introduction

Females may enhance the resistance of their offspring to infectious diseases by mating with disease‐resistant males (Hamilton–Zuk hypothesis) (Hamilton & Zuk, 1982), genetically compatible males (Penn *et al*., 2002) or multiple males (Jennions & Petrie, 2000). Numerous studies have investigated the Hamilton–Zuk hypothesis (Hamilton & Poulin, 1997; Møller *et al*., 1999), and a recent study provides direct experimental support. When female house mice (*Mus musculus*) are experimentally mated with their preferred males, their offspring are better able to survive an experimental *Salmonella* infection compared to offspring sired by nonpreferred males (Raveh *et al*., 2014). Yet, when female house mice are allowed to select their mates, they often mate with multiple males. For example, when females can choose to mate between one or two males, behavioural observations indicate that all females mate multiply (Rolland *et al*., 2003), and paternity analyses show that 29–46% of litters are multiply sired (Thonhauser *et al*., 2013b, 2014; Manser *et al*., 2015). Moreover, in wild populations of house mice, the frequency of multiple paternity ranges between 6% and 43% [mean 30%: *M. musculus domesticus* (Dean *et al*., 2006; Firman & Simmons, 2008c); *M. musculus musculus* (Thonhauser *et al*., 2013c)]. Several adaptive hypotheses have been proposed for multiple male mating (genetic polyandry) (Zeh & Zeh, 1996, 1997; Jennions & Petrie, 2000; Hosken & Stockley, 2003; Simmons, 2005), including the good genes and compatible genes hypotheses, which predict that paternity can be biased towards a high quality or a more compatible mate through sperm competition or cryptic female choice (Madsen *et al*., 1992; Tregenza & Wedell, 2002; Hosken *et al*., 2003; García‐González & Simmons, 2005; Fisher *et al*., 2006; Firman & Simmons, 2008a,b). In house mice, it has been shown that polyandry can facilitate inbreeding avoidance (Firman & Simmons, 2008b), enhance pup survival (Firman & Simmons, 2008a; Auclair *et al*., 2014) and reduce litter loss due to genetic incompatibilities (Manser *et al*., 2015; Sutter & Lindholm, 2015). Polyandry can also potentially provide genetic benefits by enhancing offspring diversity (Yasui, 1998), which may enhance resistance to infectious diseases (Jennions & Petrie, 2000). Surprisingly, however, no studies to our knowledge have tested whether multiple paternity affects offspring resistance to infectious diseases in any vertebrate species.

By increasing offspring genetic diversity, polyandry may provide two nonmutually exclusive types of indirect fitness benefits. First, it may increase the variation in fitness within while reducing the variation in fitness between litters, thereby enhancing the geometric mean fitness of females (bet‐hedging hypothesis) (Yasui, 1998; Fox & Rauter, 2003). Bet‐hedging can be selectively favoured (even if the arithmetic mean fitness of females is reduced) when environmental conditions are unpredictable, as increased genetic diversity ensures that at least some genotypes fit the prevailing circumstances. Second, increased genetic diversity within litters may enhance the arithmetic mean fitness of females (non‐bet‐hedging hypothesis). Such effects could be due to reduced competition and more effective resource utilization among half‐siblings or genetically based differences in disease resistance (Hamilton, 1987; Jennions & Petrie, 2000). For example, increased genetic diversity within insect colonies (either generated by increased multiple mating of queens or mixing broods from diverse backgrounds) reduced the intensity and prevalence of parasitic infection of colonies (Shykoff & Schmid‐Hempel, 1991; Liersch & Schmid‐Hempel, 1998; Baer & Schmid‐Hempel, 1999; Tarpy, 2003; Tarpy & Seeley, 2006; Seeley & Tarpy, 2007). It has been shown that genetic diversification provides disease control (Fuchs & Schade, 1994; Zhu *et al*., 2000; Hughes & Boomsma, 2004), and similarly, multiple male mating may allow females to increase the genetic diversity and disease resistance of their litters (McLeod & Marshall, 2009; Soper *et al*., 2012).

In wild house mice (*M. musculus musculus*), multiple‐sired litters have higher genetic diversity than single‐sired litters (Thonhauser *et al*., 2013c), but it is unknown whether increasing offspring genetic diversity provides disease resistance or any other indirect fitness benefits. Multiple mating in mice may depend upon the variation in quality of potential mates. Scent marking in mice is a secondary sexual trait that indicates male health and other aspects of quality (Kavaliers & Colwell, 1995; Penn *et al*., 1998; Zala *et al*., 2004). Interestingly, when female mice are able to select their mates, they are more likely to produce multiple‐sired litters when their potential mates show similar levels of scent marking, whereas they are more likely to produce single‐sired litters, fathered by the high‐marking male, when their available mates differ in their scent marking (Thonhauser *et al*., 2013a, b). The relationship between female multiple mating and parentage is not known for house mice, but it was suggested that females mate multiply when they detect no difference in male quality, or alternatively females may generally mate multiply and single paternity may be due to sperm competition or cryptic female choice. To understand the adaptive functions of multiple male mating, the fitness consequences of polyandry need to be evaluated – especially under infection, competition or other ecological challenges.

The aim of this study was to compare the disease resistance of single‐ versus multiple‐sired litters produced by female house mice (*M. musculus musculus*) that were allowed to choose to mate with either one or two males (Thonhauser *et al*., 2013b). We investigated how the resulting offspring were able to clear (infection resistance) and cope (infection tolerance) with an experimental infection of *Salmonella enterica* serovar *Typhimurium. S. Typhimurium* is an enteric mouse pathogen that can cause medium to severe infections in hosts (Penn *et al*., 2002) as there is much variation in the virulence of different *Salmonella* strains (from benign to fatal). Infection resistance and tolerance to this pathogen are controlled by several genetic loci (Roy & Malo, 2002), including the highly polymorphic genes of the major histocompatibility complex (MHC) (Penn *et al*., 2002), and genetic homozygosity and inbreeding influence prevalence and reduce individual resistance of *Salmonella* infection (Penn *et al*., 2002; Ilmonen *et al*., 2008b). We infected mice with two strains of *Salmonella enterica* (a strain having very low virulence and another with higher virulence) and monitored changes in health (body mass) over 17 days and then measured pathogen loads to assess the ability to control infection (resistance/susceptibility). If multiple paternity functions as bet‐hedging, we predicted reduced variance in health and pathogen loads among multiple‐ versus single‐sired litters, although no differences in the mean load between the litters. If polyandry provides fitness benefits through a non‐bet‐hedging mechanism, then the mean *Salmonella* load should be lower in multiple‐ compared to single‐sired litters. On the other hand, if females mate singly when they can choose between high‐ and low‐quality males and mate preferences function to enhance offspring resistance or tolerance to infection (Raveh *et al*., 2014), then offspring from single‐sired litters may be equal or even more disease resistant than offspring from multiple‐sired litters. In addition, we compared the resistance of the sexes to *Salmonella* infection, as there have been only few such studies in wild‐derived house mice (Zala *et al*., 2004, 2008; Ilmonen *et al*., 2007, 2008b; Raveh *et al*., 2014).

## Materials and methods

### Experimental animals and housing

Experimental mice were second‐generation descendants of wild‐caught house mice (*Mus musculus musculus*) bred for a previous experiment, in which females were released into separate enclosures and could freely choose to mate with either one or both of two neighbouring males that were restricted to their own territories (Thonhauser *et al*., 2013b). We controlled for male harassment by providing a shelter within each male territory that was only accessible for females. We did not record female mating behaviour and conducted genetic paternity analyses to identify single‐ and multiple‐sired litters. We selected 15 single‐ and 15 multiple‐sired litters (213 offspring) from 30 different females. Experimental animals originated from 26 different families. Single‐sired litters were produced by 15 dams (all except three were unrelated to each other) and 15 sires (all except four were unrelated). Multiple‐sired litters were produced by 15 dams (all except three were unrelated) and 30 sires (all but nine were unrelated). Sires were always unrelated to dams. Litter size varied from three to eleven offspring and did not differ significantly between single‐ and multiple‐sired litters (*t*‐test: *t *=* *0.562, *df* = 28, *P *=* *0.579). Also, the sex ratio of offspring (the number of males and females) did not differ between single‐ and multiple‐sired litters (chi‐square test: *χ*² = 0.004; *P *=* *0.949). Weaning occurred at the age of 21 ± 1 day and individuals were housed individually (type II mouse cages, 26.5 × 20.5 × 14 cm) under standard conditions (12‐h:12‐h light/dark cycle). Two d prior to infection, all experimental animals were moved into a separate room and placed individually into type IIL mouse cages (36.5 × 20.5 × 14 cm) with filter hoods on top. All cages were equipped with wooden bedding (ABEDD), wood shavings, a nest box and food (Altromin rodent diet 1324) and water *ad libitum*. Filter hoods were used to prevent mice from being exposed to other potential pathogens and to avoid the spread of the infection. At the start of the experiment, animals were between six and 9 months old.

### *Salmonella* infection

We measured prevalence and resistance to infection by assessing individuals' ability to control and resolve an experimental infection of a mouse pathogen, *Salmonella enterica* serovar *Typhimurium*. Although there have been numerous studies on *Salmonella* infection in laboratory mice, there have only been a few studies on wild or wild‐derived house mice (Zala *et al*., 2004, 2008; Ilmonen *et al*., 2007, 2008b; Raveh *et al*., 2014). All experimental animals were infected with two different strains of *Salmonella*: a primary infection with an avirulent strain (AroA) and 10 day later a secondary infection with a more virulent strain (LT2). The primary infection ensured that all of the mice had been exposed to *Salmonella* and provided protection against the more virulent strain (Hoiseth & Stocker, 1981; Hormaeche *et al*., 1995). The bacteria from both strains were stored as slants at 4 °C (originated from frozen stocks at −80 °C) and cultured in 7.5 mL of heart–brain infusion at 37 °C (for 13 h while shaking at 170 rpm). We diluted the cultures with sterile phosphate‐buffered saline (PBS) solution until the desired concentration of 10^4^ colony‐forming units (cfu) mL^−1^ for AroA and 10^3^ cfu mL^−1^ for LT2. To verify inocula dosage, we used quantitative plate counts (three plates per dilution). All animals received intraperitoneal (IP) injections of 200 μL AroA inoculum and after 10 days 200 μL of LT2 inoculum. Due to space and time limitations, we divided the experimental animals into 15 groups, each containing one single‐ and one multiple‐sired litter. This design ensured that mice were all infected for the same duration (17 day for both strains). Fresh inocula were prepared for both infections in each group. AroA inocula concentrations varied from 4.8 × 10^4^ to 1.0 × 10^5^ and LT2 inocula from 8.2 × 10^2^ to 2.1 × 10^3^ between groups. All experimental animals were euthanized 7 days after the LT2 infection using CO_2_. The health condition of all mice was checked daily by visual inspection.

### Assessing prevalence, resistance and tolerance to infection

To assess prevalence and the ability to control and clear *Salmonella* infection (infection resistance), we measured the number of viable bacteria in the spleen (also known as ‘pathogen load’) following previous methods (Penn *et al*., 2002; Zala *et al*., 2004; Ilmonen *et al*., 2007, 2008b). Spleens from euthanized animals were immediately removed, weighed and homogenized (Dispergierstation, T 8.10, IKA^®^‐Werke) in 1 ml phosphate‐buffered saline (PBS) under sterile conditions. We plated 50 μL of spleen homogenates and their 10^−1^ and 10^−2^ dilutions on selective agar plates (Salmonella‐Shigella Agar, Roth) and incubated the plates for 18 h overnight at 36 °C. Pathogen loads per spleen were determined by quantitative plate counts using the mean of two replicate plates for each dilution. We confirmed that pathogen load was not affected by the variation in cfu in inocula (LMM: *F*_1,28_ = 0.010, *β *= 0.00006, *SE* = 0.0006, *P *=* *0.921). To estimate individual tolerance (the ability to cope with infection among mice with similar resistance), we measured individual body mass before each infection and directly after euthanasia to calculate body mass changes over the course of the experiment and to relate these changes to individual infection rates.

### Statistical analyses

A Spearman rank correlation test was performed to assess the relationship between spleen mass and individual body mass and spleen mass and pathogen load. Differences in pathogen load among all litters were determined with a Kruskal–Wallis test, and the variance in pathogen load among all litters was compared with a Brown–Forsythe test. To test for differences in pathogen loads between single‐ and multiple‐sired litters, a Mann–Whitney U‐test was applied on the mean pathogen loads of single‐ and multiple‐sired litters. To compare the between‐litter variation in single‐ versus multiple‐sired litters, a Brown–Forsythe test was run on the mean values. To compare the within‐litter variation in single‐ and multiple‐sired litters, the standard deviation (SD) in pathogen loads of single‐ and multiple‐sired litters was compared with a Mann–Whitney *U*‐test. To assess which variables influence individual pathogen load, we applied a general linear mixed‐effects model (LMM) with individual pathogen load as the dependent variable, paternity (single or multiple) and sex as fixed factors, inocula dosage, individual body mass, body mass change during the experiment and age at the beginning of the experiment as a covariate. Family ID was included into the model as a random factor to control for nonindependence of individuals within families. The data were highly skewed and model residuals were not normally distributed. Log transformation was not sufficient to normalize residuals, and thus, we used a Box–Cox transformation to further improve the normal distribution of residuals. All statistical analyses were performed using ‘R' (version 2.14.1) (R Development Core Team, 2011). We implemented linear mixed‐effects models using the ‘lme’ function of the ‘nlme’ package (Pinheiro *et al*., 2012). We applied a backward stepwise removal procedure (Grafen & Hails, 2002) to avoid problems due to the inclusion of nonsignificant terms (Engqvist, 2005), and removed variables were re‐entered one by one to the final model to obtain relevant statistics.

### Ethics statement

This study has been discussed by the Institutional Ethics Committee in accordance with Good Scientific Practice guidelines and has been approved by the Austrian Federal Ministry for Science and Research (Permit number: Zl.22/01/97/2012).

## Results

**Figure 1:**
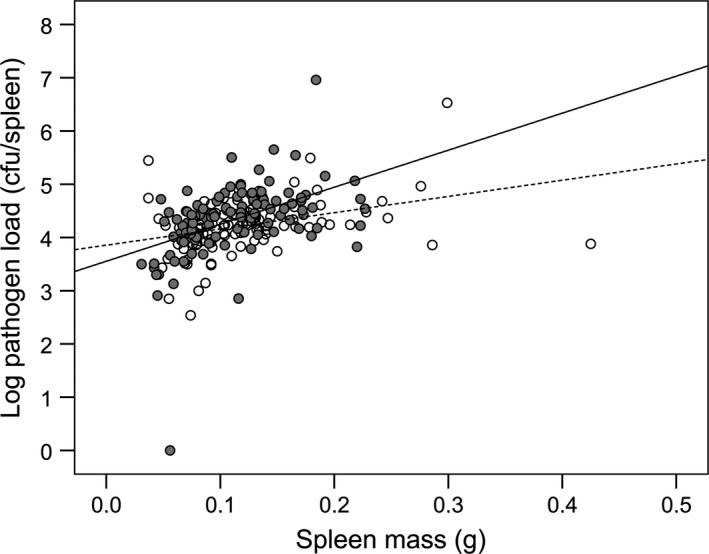
Relationship between spleen mass and pathogen load. Individual offspring spleen mass and pathogen load in single‐sired (white circles and dashed line, *R*² = 0.12) and multiple‐sired (grey circles and solid line, *R*² = 0.21) litters. Pathogen load is log‐transformed.

**Figure 2:**
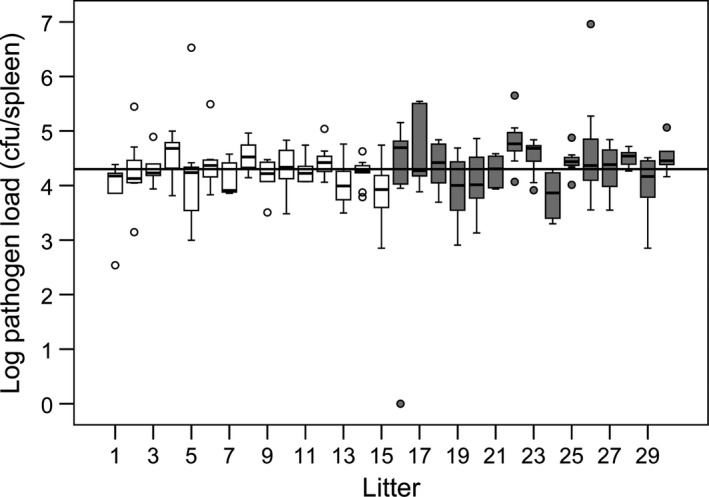
Differences in pathogen loads between litters. Single‐sired litters are depicted in white, and multiple‐sired litters are in grey. The solid line shows the median pathogen load of all litters. Pathogen load is log‐transformed.

**Figure 3:**
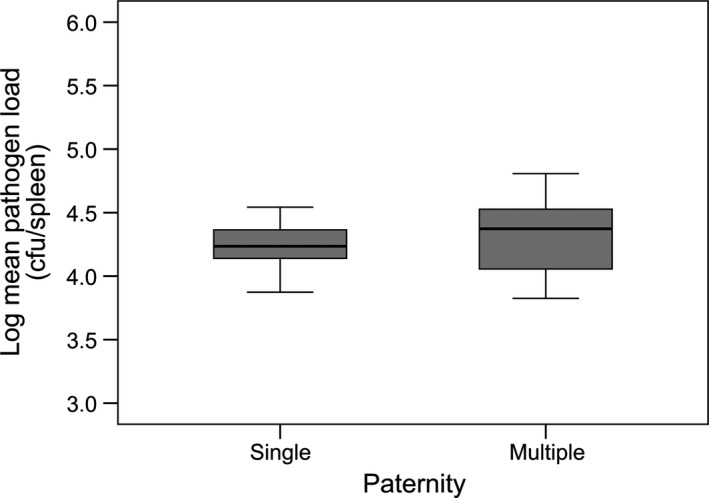
Pathogen load in single‐ versus multiple‐sired litters. Differences in the mean pathogen load of single (*N *=* *15)‐ vs. multiple (*N *=* *15)‐sired litters. Pathogen load is log‐transformed.

We found no difference in prevalence of infection among litters, as only one individual cleared the infection (all but one mouse was still infected 17 days after primary and 7 days after secondary infection). Resistance to infection was highly variable among individuals, as pathogen loads ranged from 0 to 9.1 × 10^6^ cfu mL^−1^. Spleen mass of individual mice was highly correlated with pathogen load (Spearman's rank correlation: *ρ *= 0.453, *N *=* *213, *P *=* *3.4 × 10^−12^) (Fig. 1), but not with body mass (Spearman's rank correlation: *ρ *= 0.022, *N *=* *213, *P *=* *0.748) or change in body mass over the course of the experiment (Spearman's rank correlation: *ρ *= 0.090, *N *=* *213, *P *=* *0.177). We found a significant difference in the pathogen loads among all litters (Kruskal–Wallis test: *H *=* *49.11, *df* = 29, *P *=* *0.011), but no significant difference in the variance among all litters (Brown–Forsythe test: *F*_29,183_ = 0.870, *P *=* *0.662) (Fig. 2). We found no significant difference in mean pathogen loads between single‐ and multiple‐sired litters (Mann–Whitney *U*:* W* = 94, *P *=* *0.461) (Fig. 3) and no difference in the within‐litter (Mann–Whitney *U*:* W* = 103, *P *=* *0.713) or between‐litter variance (Brown–Forsythe test: *F*_1,28_ = 0.412, *P *=* *0.527) in pathogen loads of single‐ and multiple‐sired litters.

**Figure 4:**
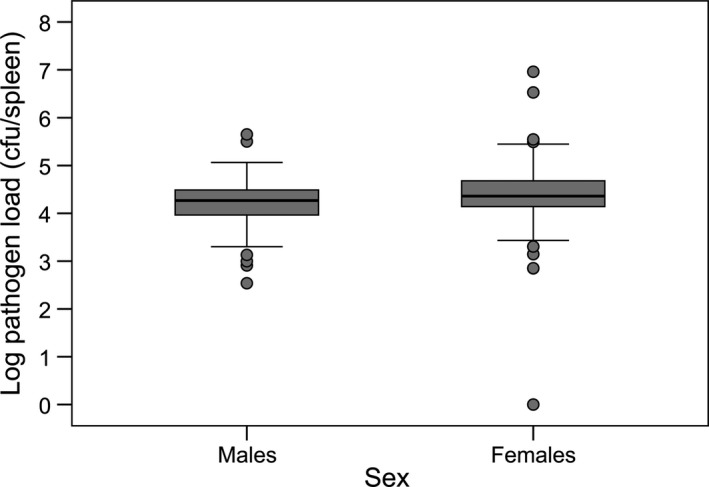
Sex difference in pathogen load. Pathogen load of males (*N *=* *108) and females (*N *=* *105). Pathogen load is log‐transformed.

**Figure 5:**
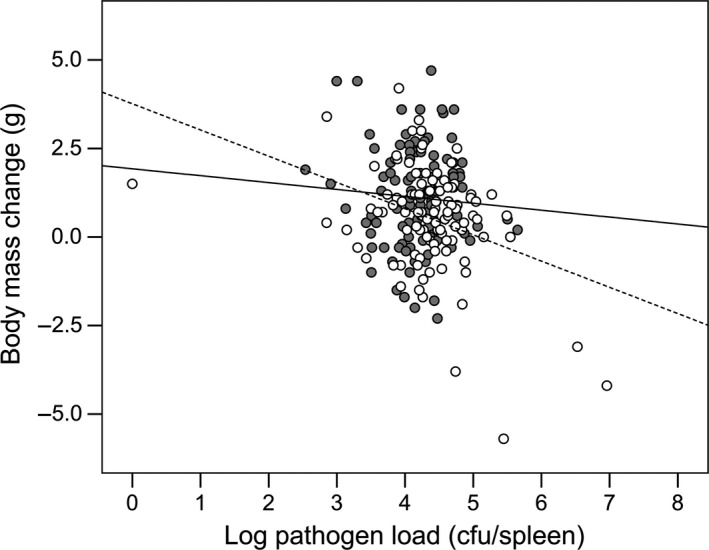
Body mass change in relation to pathogen load. Individual pathogen load and body mass in males (grey circles and solid line; *R*² = 0.005) and females (white circles and dashed line; *R*² = 0.134). Pathogen load is log‐transformed.

Individual pathogen load did not differ between offspring from single‐ vs. multiple‐sired litters (LMM: *F*_1,28_ = 1.264, *P *=* *0.271) and was not influenced by age (LMM: *F*_1,28_ = 0.220, *β *= −0.004, *SE* = 0.008, *P *= 0.643) or body mass (LMM: *F*_1,178_ = 1.197, *β *= 0.028, *SE* = 0.062, *P *=* *0.658). Interestingly, we found a significantly higher pathogen load in females than in males (LMM: *F*_1,179_ = 8.134, *P *=* *0.005) (Fig. 4) and a significant interaction between individual body mass change over the course of the experiment and sex (LMM: *F*_1,179_ = 12.974, *β *= −0.812, *SE* = 0.225, *P *=* *0.0004): female body mass significantly reduced with increasing pathogen load during infection, whereas male body mass change was not affected by pathogen load (Fig. 5). The main effect of body mass change on pathogen load was not significant (LMM: *F*_1,179_ = 1.197, *β *= −0.059, *SE* = 0.164, *P *= 0.722).

## Discussion

We found a high variation in infection resistance (pathogen loads) among individuals and a significant difference in the mean load between different litters, which is consistent with other evidence that resistance to *Salmonella* is genetically influenced. *Salmonella* is known to cause splenomegaly, and we found that spleen size increased with increasing pathogen loads, which confirms that resistance affects the pathogenicity. However, we found no difference in the mean or variance in pathogen loads between single‐ and multiple‐sired litters. Therefore, our data do not support the hypothesis that increased litter genetic diversity enhances offspring disease resistance through either a bet‐hedging or a non‐bet‐hedging mechanism in our study population. Our study may have lacked sufficient power to detect small differences in resistance, although our sample size was larger than previous studies that found genetic diversity enhances disease resistance and colony survival (Liersch & Schmid‐Hempel, 1998; Baer & Schmid‐Hempel, 1999; Tarpy & Seeley, 2006) and applying a one‐tailed test, as used in these previous studies, would not change our results. Nonetheless, our results do not necessarily rule out the possibility that multiple male mating affects offspring disease resistance for the following reasons. First, genetic diversity of litters may have been insufficient in our study to reduce the prevalence of infection. Multiple‐sired litters are more genetically diverse compared to single‐sired litters in wild populations of house mice (Thonhauser *et al*., 2013c), but we did not investigate genetic diversity in this study. In addition, multiple‐sired litters in our study had only two sires, as in wild populations (Dean *et al*., 2006; Firman & Simmons, 2008c; Thonhauser *et al*., 2013c), whereas previous studies with bees inseminated queens with sperm from 10 different drones (Tarpy & Seeley, 2006; Seeley & Tarpy, 2007). Furthermore, social insects produce larger broods than mice (honeybee queens produce up to 250,000 eggs per year), and therefore, they have greater potential to increase the genetic diversity of their broods. Second, we measured pathogen resistance against two strains of *Salmonella*, whereas the benefits of genetic diversity may only apply to more pathogens, either simultaneously or over time. In particular, bet‐hedging benefits likely only occur when hosts are infected with different pathogens having different resistance/susceptibility profiles (McClelland *et al*., 2003). Third, we assessed how mice were able to control *Salmonella* infections (pathogen load 7 days after the second infection), and although many studies have found that this measure predicts disease resistance (Roy & Malo, 2002) and survival in laboratory mice (Penn *et al*., 2002), the fitness effects of resistance are less clear in wild mice. We did not find any difference in the prevalence of infection, as only one mouse cleared the infection, and thus, further studies are needed to investigate prevalence over a longer period of time. Also, studies are needed to test the ability to tolerate and survive virulent infections (Raveh *et al*., 2014). As we experimentally infected the mice, our study design does not allow to test whether genetic diversity helps to prevent infections or reduce their spread within litters, and thus, additional studies are needed to test for such effects. Fourth, we aimed to compare the consequences of producing single‐ versus multiple‐paternity litters and we assume that females that mate multiply are more likely to have multiple paternity than other females, but studies are needed that experimentally assign paternity to control for potential differences in female quality and ensure that any benefits of multiple paternity are not masked by benefits of mate choice for a single, high‐quality male.

We found a significant sex difference in infection resistance and that males and females tolerate high bacterial loads differently, which is the first such evidence with this pathogen to our knowledge. Females had significantly higher pathogen loads (lower resistance) than males, and lower resistance (increasing pathogen loads) correlated with loss in body mass during infection in females, but not males. Thus, females were less resistant and less tolerant of infection than males. Resistance to infection is sex dependent in many species, and although there are many exceptions, males are generally more susceptible than females (Zuk & McKean, 1996). Previous studies on sex differences in disease resistance have mainly measured immune responses to novel antigens (immunocompetence) or observed parasite burdens, but our findings emphasize the importance of measuring hosts' ability to cope with infection (Schneider & Ayres, 2008). There have been numerous experimental *Salmonella* infection studies on laboratory mice (Mittrücker & Kaufmann, 2000), as they are the most common host‐pathogen model for typhoid fever, yet surprisingly few studies have examined sex differences. Early studies on laboratory mice detected no sex differences in pathogen loads (Plant & Glynn, 1976), but since then, most studies focus on one sex or the sexes are not reported. There have been only a few studies on *Salmonella* resistance on wild‐derived house mice, and only some of these compared the sexes. One study found sex differences in pathogen prevalence (females showed lower prevalence than males following experimental infection) (Ilmonen *et al*., 2008a) and another found interactions between sex and genetic control of resistance (Ilmonen *et al*., 2008b). Another study found that females had better survival following a primary *Salmonella* infection compared to males, but no sex differences in pathogen clearance, indicating a sex differences in tolerance to primary infection (Raveh *et al*., 2014). Taken together, these findings suggest that sex differences in resistance and tolerance to *Salmonella* depend upon whether the infection is a primary or secondary challenge. They also suggest that sex differences in *Salmonella* resistance and tolerance may be more pronounced in wild compared to inbred laboratory mice. It has been reported that, among other loci, an X‐linked locus (*btk*) controls resistance to *Salmonella* in mice through B‐cell functions (O'Brien *et al*., 1979, 1981), but it is not known whether this locus contributes to sex differences in resistance. Sex differences in resistance to infection are generally thought to be due to immunosuppressive effects of testosterone or other steroid hormones (Klein, 2000, 2004). In house mice, males are more resistant than females to some parasites, including *Toxoplasma gondii, Babesia microti, Schistosoma mansoni* and *Taenia crassips* (Klein, 2004; Morales‐Montor *et al*., 2004). The harmful effects of *T. gondii* infection depend on estradiol as ovariectomy reduced and administration of estradiol enhanced the development of tissue cysts caused by *T. gondii* (Pung & Luster, 1986). Estradiol reduces CD8+ T cells (Boll & Reimann, 1996), and CD8+ T cells control protection against virulent *S. Typhimurium* (at least in secondary infections) (Mastroeni *et al*., 1992; Mittrücker & Kaufmann, 2000). Also, mice deficient in CD8+ T cells that survived an attenuated *S. Typhimurium* infection were more susceptible to a virulent *S. Typhimurium* strain compared to control animals (Lo *et al*., 1999). Given that testosterone enhances CD4+ and CD8+ T cells and CD4+ T cells are also highly important in protection against Salmonella infection (Mittrücker & Kaufmann, 2000), the sex difference in *Salmonella* load in our study may be explained by differences in steroid hormone concentrations.

In summary, we found significant differences among litters in their resistance to *Salmonella* infection, which is consistent with genetic influence on immune resistance, but we found no evidence that multiple paternity affects pathogen prevalence or immune resistance to this pathogen. Increasing the number of sires might provide different results, but because the number of sires only rarely exceeds two in litters of wild house mice, previous findings from insects may not apply to mice or other vertebrates. Future studies are needed to investigate resistance and tolerance to infection in wild mice and to test the effects of a variety of different parasites on multiple‐ vs. single‐sired litters. We also found that females were less resistant to secondary *Salmonella* infection than males. This result was unexpected because males are usually more susceptible to infectious diseases than females, and because numerous studies on *Salmonella* infection with laboratory mice have not reported such sex differences.

## Conflict of interests

The authors declare that they have no conflicts of interest.
